# Applying Sensor-Based Technology to Improve Construction Safety Management

**DOI:** 10.3390/s17081841

**Published:** 2017-08-11

**Authors:** Mingyuan Zhang, Tianzhuo Cao, Xuefeng Zhao

**Affiliations:** Department of Construction Management, Dalian University of Technology, Dalian 116000, China; myzhang@dlut.edu.cn (M.Z.); chenfucc@mail.dlut.edu.cn (T.C.)

**Keywords:** construction, safety management, sensor-based technology, sensors

## Abstract

Construction sites are dynamic and complicated systems. The movement and interaction of people, goods and energy make construction safety management extremely difficult. Due to the ever-increasing amount of information, traditional construction safety management has operated under difficult circumstances. As an effective way to collect, identify and process information, sensor-based technology is deemed to provide new generation of methods for advancing construction safety management. It makes the real-time construction safety management with high efficiency and accuracy a reality and provides a solid foundation for facilitating its modernization, and informatization. Nowadays, various sensor-based technologies have been adopted for construction safety management, including locating sensor-based technology, vision-based sensing and wireless sensor networks. This paper provides a systematic and comprehensive review of previous studies in this field to acknowledge useful findings, identify the research gaps and point out future research directions.

## 1. Introduction

The development of sensor-based technologies has greatly improved information collection, data transmission and processing, which can serve as the foundation of the modernization of construction safety management. After nearly two decades of development, sensor-based technologies have facilitated the transformation from experimental exploration to practical applications. The applications of sensor-based technology in construction safety management have become the focus of current research.

Since the various safety risk factors are endless in many aspects of building construction, construction safety management includes a very broad range of topics. It is hard to give a very clear definition and scope for construction safety management. Some researchers have tried to divide construction safety management into a pre-construction stage and a construction stage [1]. In the former stage, potential safety hazards are usually identified based on experts’ or managers’ experience and eliminated through necessary preventive actions. In the latter stage, accidents are prevented by monitoring workers, machinery and the whole environment on site. Nevertheless, in the process of exploring more effective safety management methods with enhanced safety concept, it is realized that construction safety management should not be limited to merely the construction phase, but gradually proceed during in the full building life cycle, conducting a comprehensive and thorough safety management. Hence, based on the application range of different kinds of sensor-based technologies in the field of construction, this paper divides construction safety management into six aspects in detail, including accident forewarning systems, safety route prediction and planning, hazard identification, etc.

The sensor-based technology applied to construction safety management consists of sensor-based location, vision-based sensing, and wireless sensor networks, etc. The combination of multiple sensor-based technologies basically meets the technical requirements in the safety management of construction projects, which will be further discussed in Section 4. Furthermore, the intelligence of sensor-based technology helps construct an interactive management platform, which is the integration of hardware and software for data processing, significantly improving the construction site monitoring capacity and providing guarantees for construction safety.

In practice, hardware and software in data processing are the two major factors restricting sensor-based technology. Along with the increasingly in-depth research and advanced experience of utilizing sensor-based technology in some countries and regions, it is not hard to realize that the development of sensor-based technology often falls into the contradiction between the aforementioned restrictive factors. In actual use, managers usually allocate limited resources to only one of the two respects, depending on which one can lead to larger gains. Under the circumstances, stressing the one-sided technical advantages and neglecting its defects inevitably weaken the practical performance of sensor-based technology in construction safety management. How to seek a proper balance between hardware and software in data processing is vital for promoting any sensor-based technology.

Currently, utilizing sensor-based technology to improve construction safety management has been a fast-developing area as well as a great subject of interest within the engineering and academic communities. Nevertheless, there is a lack of a systematic review of sensor-based technology applications for construction safety management. Therefore, this paper aims at contributing to an in-depth investigation and providing a systematic and comprehensive review of previous studies to help researchers and managers acknowledge useful findings and capture future trends.

The remainder of the study is structured as follows: in the second section, a two-stage literature selection method was accomplished, identifying relevant papers and compiling a database of the findings. Data analysis was then performed to identify useful research findings. In light of the results from the database, the third section presents an introduction to sensor-based technologies, including sensor-based locating, vision-based sensing, and wireless sensor network. The fourth section carries out a comprehensive and systematic overview of sensor-based technology applications for construction safety management from eight aspects. The fifth section points out the direction for future work, which offers potential opportunities for researchers to conduct more relevant studies and effective measures, in order to ensure a safe construction site. Finally, the conclusions are stated.

## 2. Methodology 

### 2.1. Literature Search and Database Construction

The paper adopts a methodological approach to conduct a comprehensive review of sensor-based technology applications in construction safety management. It provides a foundation for exploring useful findings and identifying gaps for future research on the basis of previous studies. With the help of scientific selection methods, key papers relevant to the chosen topic were selected and compiled to construct a database. Data analysis was then performed from chronological and thematic perspectives in order to identify useful research findings.

A two-stage literature selection method after Tsai and Wen [2] was applied to collect key relevant papers. The time range was determined to be from 2005 to 2016. The reason is that before 2005, few sensor-based technologies were utilized in construction safety management and their application range was quite restricted. Meanwhile, the level of technology during this period was much too simple, and the content of relevant papers would not play a positive role in improving construction safety management. As for the selection of sensor-based technology, it is conducted in the following steps: (1) based on the review of relevant papers, some sensor-based technologies were preliminarily identified since their utilization was pertinent to improve construction safety management; (2) for those technologies with little literature available (only 1 or 2 references) that only stated their capability of improving construction safety management without verification and test measures (e.g., laser scan and infrared), or those combined with other sensor-based techniques, not playing a central role but only providing supplementary support for construction safety management (e.g., applying Bluetooth to transmit data), should be removed. Thus the sensor-based technology consisted of sensor-based location, vision-based sensing and wireless sensor networks. The preset topic was the application of sensor-based technology in construction safety management. In the first stage of literature selection, the Web of Science was chosen as the primary literature source and the Engineering Index was selected as a supplement.

A comprehensive literature search within SCI database was implemented using the “Title/Abstract/Keyword” field. The keywords were chosen as ‘GPS’ OR ‘RFID’ OR ‘WLAN’ OR ‘ultra-wideband’ OR ‘Zigbee’ OR ‘ultrasound’ OR ‘camera’ OR ‘sensor’ OR ‘wireless sensor network’ AND ‘construction’ AND ‘management’ AND ‘safety’ OR ‘hazard’ OR ‘incident’ OR ‘accident’. A total of 156 related papers were found and transferred into the second stage of literature selection, which serves as a refinement. First, 11 papers were removed due to duplication. Considering the publication types, 42 papers belonging to “Patent”, “Editorial”, “Book”, “Letter”, “Report” and “Case Report” were then removed. In addition, 19 papers did not match the preset topic, were also removed. For example, exploiting RFID and UWB’s data transmission capacity to conduct construction safety management rather than their positioning ability, which is not relevant to the preset topic and thus needs to be removed. A total of 84 papers with specific requirements were acquired from the SCI database.

**Figure 1:**
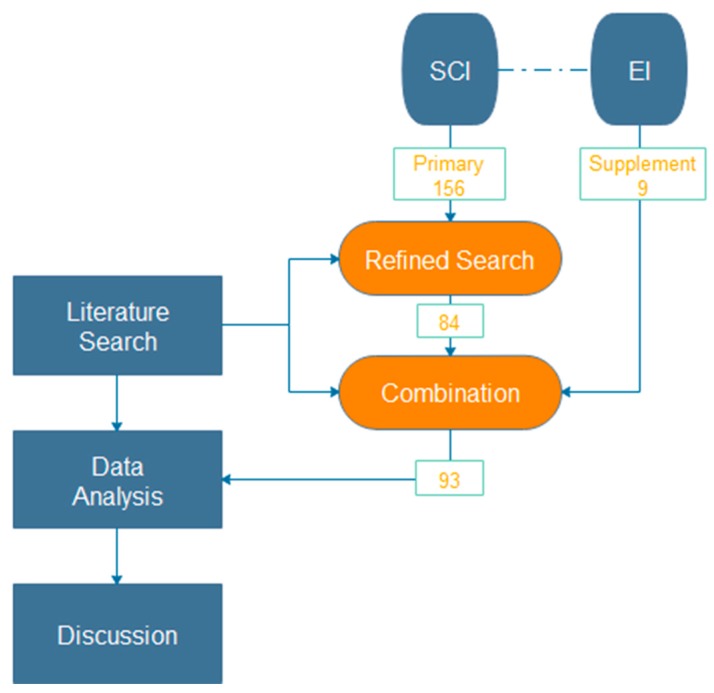
The process of literature database construction.

After the two-stage literature selection, it was found that there were a few papers retrieved from SCI database on using locating sensors for real-time underground positioning in unstable and unpredictable construction environment such as tunnels and mines. Since this part is one of the important points of Highly Dangerous Operation Management, it is necessary to supplement it through an EI search. The method of selecting papers from EI was the same as SCI search. Nine articles of satisfactory quality were selected. Overall, 93 papers were finally obtained by selection and supplement, which built the desired database. The process of database construction is shown in Figure 1.

### 2.2. Results and Discussion

#### 2.2.1. Year Profile of Publications

**Figure 2:**
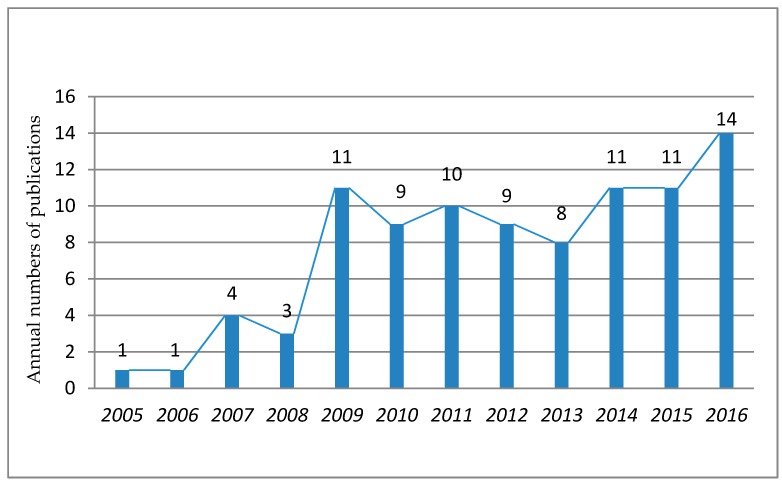
Year profile of publications.

The annual publication dates of the 93 articles are shown in Figure 2. Before 2009, no more than four publications per year were related to construction safety management based on sensor technology. A sharply increasing number of related papers have been published since 2009. Though there are fluctuations in publication quantities, the annual number reaches an average of nine. During the same period of time, the RF locating sensor-based techniques (e.g., RFID, UWB) and the wireless sensor network became well developed and gradually applied to construction safety management. The increased publication number indicates that the application of sensor-based technology is getting more and more attention from researchers in construction safety management and has become an important part of construction management practices.

#### 2.2.2. Application Trends of Sensor-Based Technology

**Figure 3:**
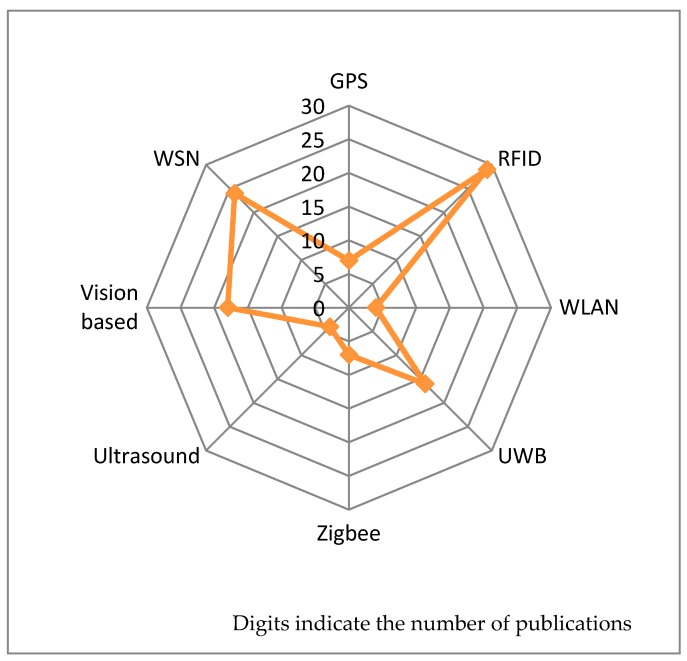
Application trends of sensor-based technology.

The distribution of sensor-based technology applied in construction safety management is shown as Figure 3. The most widely used technique is RFID, with which 29 studies are conducted. Wireless sensor networks (WSNs) ranked second (24 times), followed by vision-based sensing (18 times) and UWB (16 times). The least used techniques are WLAN and ultrasound, with four papers each. By percentage, RF locating sensor-based technologies (RFID and UWB) account for 41.28%, followed by sensors and WSN (22.02%) and vision-based sensing (16.51%). Among these three sensor-based technologies, sensors and WSNs are an advanced technology which is in the transitional stage from development to maturity, while RF locating sensor-based technology is the most mature and widespread technique, and it consequently attracts more attention. Though vision-based sensing is a traditional technique, it has the potential to be applied to brand-new fields. The reason is that with the development of machine learning and computer vision in image processing, the information in the images or videos, which cannot be identified by professionals, can be read and understood by specific algorithms instead. Compared with the past condition, these applications are brand-new fields and helpful to enhance construction safety management. Therefore, researchers are still devoted to exploiting this technique.

#### 2.2.3. Distribution of Research Topics

**Figure 4:**
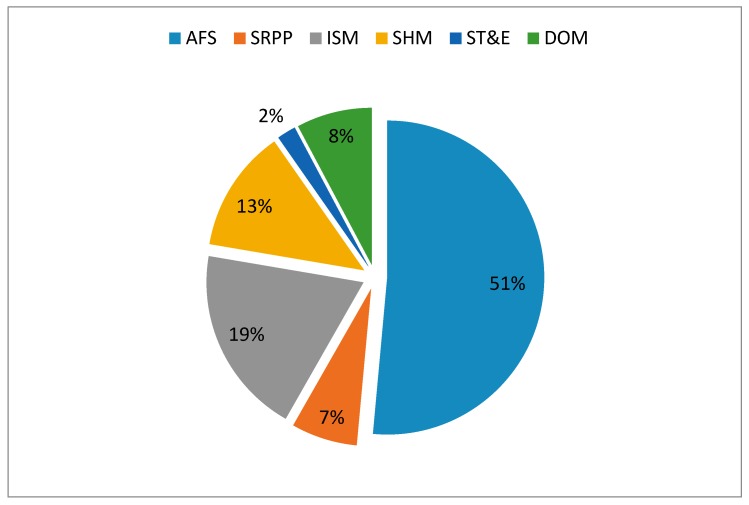
Distribution of research topics.

The research topics are summarized through analysis and classification of 91 papers, including accident forewarning system (AFS), safety route prediction and planning (SRPP), integrated safety management (ISM), structural health monitoring (SHM), safety training and education (ST&E) and highly dangerous operations management (DOM). The distribution of research topics is shown in Figure 4. The most widely studied research topic is AFS with 53 papers involved, accounting for 51% among all research topics. ISM is ranked second with 20 occurences, accounting for 19%. SHM occupies the third place with 13 referencess, accounting for 13%. In contrast, the three least studied research topics are ST&E, SRPP and DOM, which cumulatively account for 17%.

## 3. Overview of Sensor-Based Technology

### 3.1. Locating Sensor-Based Technology

#### 3.1.1. GPS

GPS, namely the global positioning system, consists of satellites, ground control stations and user receivers. Owing to its capacity of providing 3D coordinates including points, lines and planes in a fast, accurate and efficient way under all-weather circumstances, it has been widely utilized in different fields, e.g., geodesy, photogrammetry, marine surveying and mapping. GPS has also been promoted greatly in construction safety management in the last few decades. Besides its uses in engineering surveys and monitoring the deformation in buildings or building components, it has been developed in safety monitoring of building construction, including machinery equipment and construction materials.

GPS is suitable for tracking objects in outdoor environments, however, it does not work well indoors with obstacles such as basements, tunnels, culverts, etc. The accuracy and efficiency decrease evidently once the signals are blocked in such conditions. Lu et al. [3] pointed out that the average error in tracking a concrete mixer truck, in a large dense urban area in Hong Kong, was less than 10 m using a combination with GPS, dead reckoning vehicle navigation and Bluetooth beacons. Pradhananga and Teizer [4] reported an average error of 1.1 m when locating equipment with GPS in an open area, but it increased to 2.15 m and 4.16 m in the presence of nearby obstacles.

#### 3.1.2. RFID

RFID is short for radio frequency identification, which identifies a specific target through radio signals. It can read and write corresponding data without mechanical or optical contact with the identification system. RFID consists of tags, readers and antennas [5]. Since it is able to locate single or multiple targets precisely in static or dynamic indoor environment, RFID has been widely used in construction safety management, such as AD, HI, ISM and AFS. Song et al. [6] found that the average error of 2D positioning with RFID was 3.7 m, which was similar to Gu’s report [7]. The experiments conducted by Razavi and Moselhi [8] showed the average positioning error was about 1.3 m in indoor environments. In practice, the accuracy of RFID can be further improved by promoting relevant algorithms or adopting different locating methods [9].

#### 3.1.3. WLAN

Wireless local area network (WLAN) is a data transition system using RF technology. WLANs can access the network in any location within the coverage area of wireless signals and calculate the target’s position from the strength of the detected signal. The positioning system based on WLAN requires deployment of wireless signal transmitters, and the target must be in the signal coverage area, thus limiting its usability in the dynamic and complicated construction site. In practice, the obstacles may hinder or even reflect the electromagnetic signals, affecting the WLAN’s positioning accuracy, and restrict the development of WLAN in construction site. Khoury and Kamat [10] tested the accuracy of WLAN positioning system in the laboratory, showing an average error of 2 m. Taneja et al. [11] reported that the positioning error ranged from 1.5 m to 4.57 m with a credibility level of 95% for static targets and about 7.62 m for dynamic ones with a credibility level of 95%.

#### 3.1.4. UWB

Ultra-wideband (UWB) is a wireless positioning technique newly-developed in recent years. It has a good application potential in the field of wireless indoor positioning. UWB takes advantage of ultra-wideband signals that are suitable for high-speed and short-range wireless transition due to their wide spectrum range [12]. Compared to other narrow-band transition systems, it is less susceptible to multipath interference, thus it has the capability of real-time tracking for multiple targets with high sampling speed, high accuracy and low energy consumption [13].

UWB has been well accepted by scholars and construction managers and gradually popularized in related experiments and practices. So far, it has been utilized in fields including AP, SD, HI and ST&E. In general, the average positioning error is about 0.5 m [14] and the accuracy can reach the centimeter level in indoor environment. Maalek and Sadeghpour [15] reported that the 2D positioning accuracy was 20 cm and 40 cm for 3D positioning in open area with 70% credibility level for both. In contrast, Cheng et al. [16] reported the UWB was much less accurate in a large area (65,000 m^2^) affected by the frequency of positioning labels. A set of tests showed the positioning accuracy of UWB was 1.26 m with 1-Hz label and 1.63 m with 60-Hz label. In addition, the obstacles in work environment and metal interference will have a significantly negative impact on UWB’s positioning accuracy [14].

#### 3.1.5. Zigbee

Zigbee is a two-way wireless communication technique with the characteristics of short distance, low complexity, low energy consumption, low transition speed and low costs [17]. It is mainly used for data transition among various electronic devices. Zigbee is widely favored by the researchers in China and in recent years is becoming a hot technique for conducting DOM in locations such as tunnels, roadways and underground mines. On the other hand, scholars from other countries have explored its application potential by combining it with other positioning techniques such as RFID and WSN rather than the use of Zigbee alone in AP, AFS, etc. Meng et al. [18] reported an average error of 0.76 m when acquiring personnel position data in coal mines. Shen et al. [19] designed an automated tunnel-boring-machine positioning system based on Zigbee and tested its performance. The test was conducted by the designed system and a specialist surveyor independently. The differences between the two surveying were less than 2 mm, verifying the accuracy of the designed system.

#### 3.1.6. Ultrasound

An ultrasound positioning system uses sound speed and transfer time to calculate the distance between the measured point and a fixed point, and identify the target with triangle location method. The accuracy can usually reach centimeter level and the technology is mature and low cost. However, ultrasound positioning systems have some limitations. For example, the quick attenuation of ultrasound in air restricts its transition distance; it cannot penetrate obstacles such as walls and can be easily distorted by the reflected signals caused by metal objects.

Cricket is a mature ultrasound positioning system. It requires the targets to carry signal receivers and the signal transmitters mounted on walls or ceilings. In order to deal with the insufficient number of signal transmitters, the system applies RF as a backup method to provide positioning data. Tests showed the positioning error was 10 cm and the orientation accuracy was 3 degrees [20]. Skibniewski and Jang [21] employed a combination of ultrasound and RF to track the construction material in a construction site, achieving an accuracy of less than 0.2 m with 80% credibility level ranging from 1 m to 15 m under line-of-sight conditions. Another set of experiments showed that the average positioning accuracy was 0.97 m.

### 3.2. Vision-Based Sensing

Vision-based sensing uses imaging sensors to collect photos or videos. The collected data is then analyzed with specific algorithms to perceive and understand the surrounding environment. In vision-based sensing, the target does not need to carry any device. The technique itself can meet the positioning requirements in a relatively large area. However, the vision-based sensing is also vulnerable to the impact of surrounding environment, such as lighting condition and background color [7].

In practical use in most countries around the world, including China, the application of vision-based sensing is limited to the elementary level, namely setting up video surveillance systems to transmit images or videos of various construction scenes to a surveillance center. Professionals are hired to identify useful information from images or videos and make decisions. This level is far from intelligent due to the low degree of information utilization and low accuracy of identification, leading large amount of information being unused or even ignored directly.

To resolve the dilemma in practical application, foreign research has focused on the development of algorithms to replace manual supervision so as to read and understand the useful information from images quickly and accurately. Though under some circumstances, the actual effect of some algorithms is not as satisfactory as desirable, e.g., the machine learning in image processing is not as accurate as human interpretation, the unceasingly improving algorithms and advances in technology will eventually overcome the current obstacles. Since there is a huge amount of information in images or videos neglected by humans but that can be read and understood by algorithms, it provides a foundation for the application of vision-based sensing in construction safety management, such as AP, SD, HI, ISM, etc.

### 3.3. Wireless Sensor Network

The sensors applied in construction safety management mainly include temperature sensors, displacement sensors, light sensors, optical fiber sensors and pressure sensors. They play an indispensable role in real-time monitoring of structures or structural components. Sensors usually acquire information and store data in a passive manner and cannot read and understand the collected information proactively. In practice, wireless sensor networks are a proper way to turn passivity into initiative, and have thus become one of the research hotspots in sensor applications.

#### 3.3.1. Sensors

(1) Temperature sensors

The main applications of temperature sensor include shrinkage crack monitoring for mass concrete construction [22], concrete curing [23], assisted management for winter construction and freezing method construction [24] and temperature monitoring of structural components for improving the installation accuracy.

(2) Displacement sensors

The main applications of displacement sensors include building inclination monitoring [25], building subsidence monitoring, geological prediction and geological hazard pre-warning [26].

(3) Light sensors

Light sensors are mainly used for nondestructive examination of structural components, including concrete constructions, pile foundations, welding seams in steel structures, etc.

(4) Optical fiber sensors

Optical fiber sensors are widely applied in long-term monitoring for structural safety. They are usually integrated into a WSN so as to turn the whole monitored object into a sensing structure. These sensors can be used for monitoring strains, deformations and cracks of structures, and safety evaluation for mass concrete constructions. For example, the optical fiber sensors have been used in the health monitoring and safety assessment of the Three Gorges Dam and some bridges in China [27,28].

(5) Pressure sensors

Pressure sensors are useful in structural load measurement. They have been used for monitoring roads, bridges and buildings, especially in pre-stressed engineering, testing end bearing capacity of pile foundations.

#### 3.3.2. Wireless Sensor Network

A wireless sensor network (WSN) is a set of spatially distributed sensors to monitor physical or environmental conditions and to cooperatively pass the data via a network to a main location. A WSN is usually composed of a central processor, communication module and sensor nodes with internal or external power supplies. With the help of a WSN, the system acquires local information as a whole and transits collected information to the terminal server automatically to process the collected data. In this way, the key environmental information is collected passively, but being transited and processed actively.

Owing to the application of WSN in remote monitoring of engineering structures, pressure sensors installed on vehicles can transmit real-time information of cargo-handling to the terminal server; temperature sensors embedded in construction materials can detect temperature changes to avoid the dangers of extremely high or low temperatures; displacement sensors and pressure sensors embedded in concrete structures are able to collect real-time information including stresses and strains, thus achieving long-time monitoring.

### 3.4. Summary

As for locating sensor-based technology, different technologies are suitable for different working conditions. GPS does not work well in indoor environment, because its accuracy and efficiency decrease evidently once its signals are blocked. RFID, UWB and Zigbee are all locating techniques based on radio frequency, and the arrangement of signal transmitters and receivers directly affects the effective range and accuracy. Besides, it is not cost-effective to deploy large amounts of signal transmitters and receivers to cover an entire construction site. Meanwhile, obstacles in the complicated and dynamic environment may block or interfere the RF signals, affecting positioning accuracy. Since WLAN is a locating technology based on wireless signal, it also faces the same problems caused by blocked signals in practice, but it is less expensive with relatively satisfactory positioning accuracy compared with RF location techniques. In general, ultrasound provides the most accurate results under line-of-sight conditions. However, its application is limited by its short spreading distance and the signal strength has to be high enough to reduce the impact of obstacle blockages and interference. The accuracy of different locating sensor-based technologies is summarized in Table 1.

The precise positioning capability of different locating sensor-based technologies is the prerequisite and foundation of realizing some important aspects of construction safety management, such as hazard identification and accident forewarning systems. Based on different construction site conditions and safety management requirements, in order to locate various targets such as personnel and machinery or identify dangerous zones like near-edge- opening areas, appropriate sensor-based locating technologies are selected on the basis of the needed positioning accuracy. For example, taking a concrete mixer as the tracking and positioning target, the locating system’s location accuracy should be within a few meters, considering the vehicle’s size. According to concrete mixer’s working environment, GPS or RFID will be a proper option for setting up the above system. In practice, one single locating sensor-based technology may not be able to meet the positioning requirements proposed by construction safety management, so multiple ones are usually combined in use to achieve the desired result.

In general, besides locating sensor-based technology, vision-based sensing and wireless sensor networks provide a new approach for advancing construction safety management, while the development of new and effective data processing algorithms strongly support for the promotion of construction safety management. Three types of sensor-based technologies are compared in Table 2. Commonly-used algorithms were presented in Table 3.

In Table 2, the paper proposes a qualitative way to assess the complexity of algorithm and layout based on the previous literature. In the column of algorithm complexity, the localization algorithms such as Triangulation and Time of Arrival (ToA), used in locating sensor-based technology, are relatively simple and easy to implement without much computation. The algorithms utilized in vision-based sensing technology need to achieve certain objectives, including object tracking, image matching and classification, so the principle of such algorithms is complex and the computation is usually huge. As for wireless sensor network’s algorithm complexity, it is neither low nor high, but somewhere in between, so it is deemed as “moderate”. Similarly, in the column of layout complexity, for GPS, no additional equipment is required to implement the positioning function, so the layout complexity of GPS is the lowest. On the contrary, to set up a wireless sensor network, various sensors with different functions need to be installed in advance, along with other key modules which perform data processing, data transmission and power supplying. As a result, the complexity of WAS’s layout is the highest. As for UWB, Zigbee, RFID, WLAN, ultrasound and vision-based sensing technology, the implementation of their related functions needs supplementary equipment and devices such as tags, readers, antennas and cameras, but not as complicated and diversified as a WSN’s. Therefore, the layout complexity of the above sensor-base technologies is moderate. Besides the complexity comparison of algorithm and layout, the paper also summarizes the construction environment limitations of locating sensor-based technology, vision-based sensing technology and wireless sensor network to help researchers and managers better understand the adaptability of different sensor-based technologies.

In Table 3, the commonly-used algorithms are classified according to the applications and requirements of construction safety management, including localization, object tracking, image matching, noise & error removal and classification. It is realized that some classical algorithms can be improved and modified for specific purposes, and quite a few new algorithms are being developed with the promotion of science and technology areas such as machine learning and computer vision.

**Figure 5:**
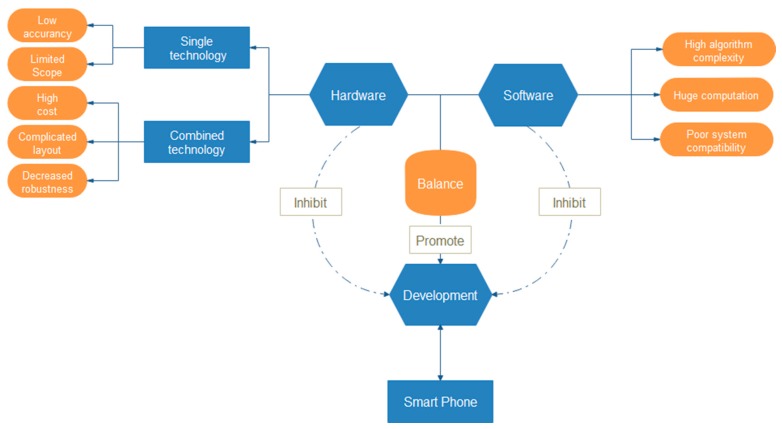
The dilemma of development of sensor-based technology.

In the process of practically conducting construction safety management, although hardware and software can both enhance the safety management level, the requirements proposed by them cannot be met at the same time based on the actual situation at the construction scene, such as costs, accuracy and complexity. In general, applying sensor-based technologies to improve construction safety management always falls into the contradiction between these two restrictive factors. On the one hand, stressing the advantages of hardware such as high utilization rate, abundant information or low costs, but ignoring its software shortages may cause multiple problems including high complexity of algorithms and huge computation burden. On the other hand, putting too much emphasis on software superiorities, e.g., accurate initial information, real-time data acquisition capability and simple data processing, but neglecting the limitation of hardware may lead to extremely complex hardware layout, high costs, system instability and low fault tolerance. The dilemma of development of sensor-based technology is shown in Figure 5.

A proper balance between the hardware and software is a key to promote sensor-based technology in construction safety management. It is vital to select hardware with moderate costs and software with easy data processing in order to acquire and process the collected information. Being a good software development platform, smartphones integrate multiple sensor-based technologies with a low manufacturing cost. From this point of view, exploiting the use of smartphones will be a good choice to promote the development of sensor-based technology in construction safety management.

## 4. Applications of Sensor-Based Technology in Construction Safety Management

**Figure 6:**
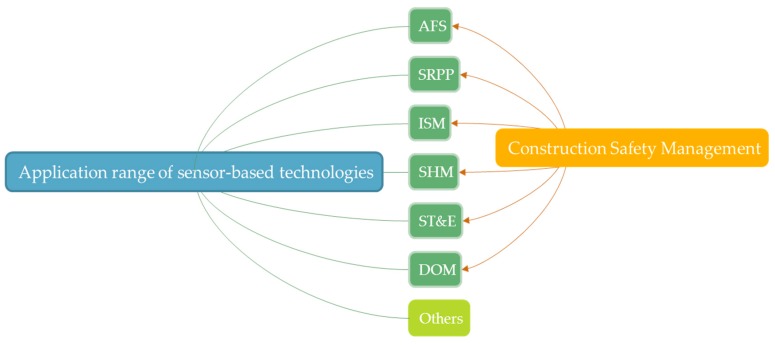
The relationship between sensor-based technology’s application range and construction safety management.

Though it is hard to give a very clear definition and scope of construction safety management, on the basis of application range of different sensor-based technologies mentioned in the previous literature, it can be narrowed down to six aspects to discuss in detail, including AFS, SRPP, ISM, SHM, ST&E and DOM. The relationship between sensor-based technologies’ application range and construction safety management is shown in Figure 6. The main applications of sensor-based technology in construction safety management are presented in Table 4.

### 4.1. Accident Forewarning System

Under the background of construction, the safety management for accidents usually adopts the approaches of “accident prevention” and “accident control”. The former is for the accidents that haven’t happened yet, while the latter is for the accidents that have happened or are happening right now. Based on the preventive theory, “accident prevention” aims at discovering and eliminating the potential factors that may cause accidents. Compared with “accident control”, “accident prevention” is obviously more effective and economical since the loss is irreversible when accidents have happened.

On the basis of the “accident prevention” idea, utilizing accident forewarning system is a proper way to deal with the potential factors that may cause construction accidents. It is deemed as an early warning system capable of complete and continuous detecting, judging and identifying unsafe behavior of personnel and machinery, and taking necessary actions such as sending alerts to construction workers or managers. In other words, it provides support for the prediction and prevention in the accident prophase as a whole.

Before applying an accident forewarning system, some preliminary work must be done to supplement and improve the “accident prevention” idea. Soungho and Tomohiro [31] collected and sorted the accident records of heavy equipment (e.g., excavators and cranes) on construction sites in order to find out the reasons for accidents. This provided a foundation in setting up an anti-collision system for heavy equipment based on RFID and clarified the key elements in the system. Similarly, Sleiman et al. [32] proposed solutions to prevent tower crane collision accidents from developing an automated planning tool based on sensing and simulation and numerical simulation, respectively.

In the accident forewarning system (AFS), the main research work focuses on three aspects: early warning index, early warning statistical method and the system applicability. With the development of early warning management theory, AFS based on sensor-based technology has made a signification breakthrough. For example, Zhou et al. [33] put forward potential factors affecting construction safety through the applications of information technology in AFS. Kim et al. [34] studied the applications of 3S (GIS, GPS and RS) technology, RFID and information technology in construction safety management, which can be extended to design the AFS. Furthermore, Wu et al. [35] and Grishma et al. [36] pointed out that the WSN based on RFID, Zigbee and other location sensor technologies had been utilized in automatic identification and prevention of construction accidents. As for machinery equipment collisions, Zhong et al. [37] adopted specific sensors to monitor the operation state of tower cranes. The collected data was transmitted to a remote monitoring platform through wireless network based on Zigbee. The anti-collision algorithm was run in the terminal located in driver’s operation room to ensure the safety of crane tower during the whole construction. Hwang and Liu [38] installed RFID labels on two tower cranes, calculated the dynamic distance between cranes and evaluated the collision probability through UWB’s real-time locating ability. Similarly, Chae and Yoshida [31] designed a system based on active RFID tags. The system was applied in the excavator safety management and its effectiveness and practicability was validated by field tests.

Aiming at reducing injuries in machinery accidents, researchers have tried to explore the solution from the dual perspectives of machinery equipment and workers, respectively. Ray and Teizer [39] devised a system which could identify a driver’s vision blind areas based on the location of his or her head. The system then alerted the driver to improve concentration and take necessary actions to prevent accidents caused by blocked sight. In order to provide real-time safety assistance to crane operators and to mitigate human errors during lifting operations, Fang et al. [40] developed a framework for real-time pro-active safety assistance for mobile crane lifting operations. The critical motions of crane parts were captured by a sensor system and the lift site conditions were modeled based on point cloud data. Field tests proved the validity of the system in making timely decisions to mitigate the risk. Park et al. [41] defined the construction machinery’s workplace as dangerous zones. When workers were too close to these areas, a designed system would send alerts to workers automatically. The field experiments tested the system performance based on RFID, Bluetooth and magnetic signals, respectively. Applying multiple sensors, ultrasound and infrared, Lee et al. [42] developed and deployed mobile sensing devices in some dangerous zones in construction site, workers would be alerted by broadcasting or text messages when they came close to the preset areas.

Because of the large occurrence of accidents involving falls from high elevations and their devastating consequence, some researchers have been devoted to promoting the forewarning system for these accidents. Carbonari et al. [43] classified areas that may have accidents arising from falling objects. The workers’ real-time location was obtained by UWB location technology, based on which the managers were able to determine whether the workers were in dangerous zones. If so, alerts would be sent to workers to stay away from dangerous areas or pay attention to falling objects. Additionally, RFID was utilized to locate workers doing high elevation work in real time [9]; a fairly complete system based on locating technologies and BIM model was proposed to warn of potential construction accidents [44].

The high frequency of false alarms has been identified as a major limitation of prevalent methods for preventing struck-by hazards. Aiming at resolving three major reasons for the generation of high false alarm rates and reducing them, Wang and Razavi [45] evaluated and compared the effectiveness of two 4D models, utilizing sensors and locating sensor-based technology, through simulation and field experiments. The developed rigorous 4D models can be employed for several types of contact collision that involve temporal and permanent site facilities, materials transported in air, and equipment and workers on foot. Furthermore, reduced false alarms will improve construction safety, productivity, and mobility.

### 4.2. Safety Route Prediction and Planning

Route planning of machinery equipment or workers in construction site is vital for construction safety management. It can not only predict the trajectory of machinery equipment or personnel, but also prevent collision accidents and avoid personnel entering the dangerous area unintentionally.

#### 4.2.1. Route Prediction and Planning for Machinery Equipment

Zhang et al. [46] proposed a location data processing method based on UWB to assist crane drivers. The labels at key positions of the crane were used to predict the crane’s route and trajectory so as to improve driver’s context awareness. Its efficiency was verified by tests in an outdoor environment. Besides locating sensor-based technology, vision-based sensing can also be utilized to predict the trajectories of machinery equipment. Yang et al. [47] used 2D and 3D pose tracking algorithms to track the trajectory of crane’s arm to predict the operation state of the crane. The field test showed that the crane’s working condition could be identified correctly.

#### 4.2.2. Route Prediction and Planning for Workers

Teizer et al. [48] tracked workers with UWB to analyze their trajectories, which laid a foundation for applications in construction safety management. Furthermore, Cheng et al. [34] conducted an in-depth analysis of workers’ trajectory based on UWB location technology. With the route planning algorithm, the probability grids of workers’ location were obtained, based on which the optimal route for workers on construction sites was proposed. Zhu et al. [49] acquired location information of workers and equipment by using multiple cameras at a construction site. The trajectories of workers and equipment were analyzed by a Kalman filter, which can predict their next locations as well. The experiments verified its prediction accuracy.

### 4.3 Integrated Safety Management

The content of integrated safety management includes two parts: quality inspection for construction materials and resources and management for workers’ health and safety.

#### 4.3.1. Quality Inspection for Construction Material and Resources

The quality of construction materials and resources has an effect on construction safety in an indirect way. On one hand, based on the opinions of construction industry experts, the hidden troubles caused by quality defects and cheating on labor and materials may lead to serious safety accidents in the operation and maintenance stages; on the other hand, quality defects can bring about quality issues such as rework and repair, leading to demolition, schedule pressure, extra working time and unstable work processes, which may all increase the risk of safety incidents. To verify this hypothesis, on the basis of empirical data collected from 32 building construction projects, ranging in scope from $50,000 to $300 million dollars, Wanberg et al. [50] tried to find out the relationship between quality performance and construction safety with empirical inquiry. Several quality metrics (e.g., cost of rework per $1 M project scope and rate of rework per 200,000 worker-hours) were used as predictor variables and first aid and Occupational Safety and Health Administration (OSHA) recordable injury rates were used as response variables. The linear regression among predictor and response variables showed that the first aid rate is positively correlated to number of defects ($r^{2}=0.548$; *p*-value =0.009) and the OSHA recordable injury rate is positively correlated to rework ($r^{2}=0.968$; *p*-value =0.032). In general, a project with a poor quality performance has a higher likelihood of injuries, which has a negative impact on construction safety. Therefore, linking construction safety to quality of material and resource provides a holistic pathway to encourage safety improvements.

Establishing a quality inspection and management system of construction materials and resources is a way to fulfill total quality management (TQM), which can lead to simultaneous improvement of both safety and quality [51]. As a significant part of the above system, the accurate real-time location of construction materials and resources can be acquired by sensor-based technology. Along with other vital information including material and resource inspection reports, certifications, arrival time and departure time, the system can not only provide information required for management work automatically and accurately, but also significantly save labor and time costs. Compared with traditional management, it solves the disordered, complicated and inefficient situation in quality inspection and management, which helps to reduce the risk of quality defection at the beginning. At present, scholars have been exploring the possibility of applying different sensor-based technologies to quality inspection and management system of construction material and resource.

The RFID technology has attracted much interest due to its technological maturity and locating ability in indoor environment. Different kinds of systems based on RFID have been developed in order to track construction materials and resources in real time, collect and analyze quality information automatically, reduce labor costs and avoid delays [52,53,54]. Combined with other sensor-based technologies, the systems can be further applied to quality inspection. Chen et al. [55] developed an automatic positioning system for precast concrete components based on RFID and GPS for the purpose of reducing incorrect installation caused by components’ misalignment. Wang et al. [56] utilized RFID, mobile devices and wireless communication network to build a concrete quality inspection system. The tests showed that the system could improve the timeliness of the quality inspection work, reduce operation costs and increase customer satisfaction. Similarly, Kim et al. [57] combined RFID with UWB to achieve effective management for busy construction sites. It was shown that the system could simplify management processes of large-scale construction projects. In addition, ultrasound [58] and vision-based sensing [59,60] were also applied in the quality inspection and management work for construction material and resource.

#### 4.3.2. Management for Workers’ Health and Safety

Over-exposure to high temperature and high humidity working environments is the direct cause of some occupational diseases, which will directly affect the personal psychological state of workers and the behavioral effects of the work [61], increasing the risk of construction safety accidents. Therefore, the management for workers’ health and safety is a crucial part of construction safety management. Similar to construction material and resources, various sensor-based technologies provide a new platform for worker health and safety management. Huang et al. [62] proposed a surveillance system (RFEMS) based on radio frequency identification tags to track and identify workers on construction sites. It was energy-saving and could constantly work for more than 124 h as proved by field experiments. The system could recognize workers’ activity patterns and laid a foundation for worker health and safety management. Furthermore, Arslan et al. [63] built a management system based on BIM and WSN to monitor construction environments in real time and conduct special management for high temperature and high humidity working conditions. The rescue and safety management of workers in emergency cases could be realized visually through this system.

### 4.4. Structural Health Monitoring

The infrastructure quality and security issue is related to the livelihood of people, and related to social stability and healthy development of the national economy. The consequences of infrastructure’s damage are potentially catastrophic. Monitoring the related indexes such as temperature, deformation, stress and displacement is a proper way to acquire health and safety condition of the infrastructure in real time. Hence, the structural health monitoring is an important part of construction safety management.

Sensors and WSN play an important role in structural health monitoring and have been widely applied to safety management of construction sites. For example, temperature sensors embedded in concrete can record the concrete hardening process in an early phase, which has been used to evaluate the quality, compressive strength and flatness of concrete [64]; optical fiber sensors embedded in concrete have been applied to monitor the strain and cracks of structures [65]. In recent years, WSN has been widely utilized in structural health monitoring of bridges [66,67,68,69,70,71], dams [72,73] and other important infrastructures concerning people’s life. For example, Shen et al. [74] developed a WSN for structural health monitoring of China National Stadium (Bird’s Nest). The network was composed of 290 sensors, measuring stress, displacement, acceleration, wind speed and temperature. The data collection lasted for more than a year and the results were satisfactory. Furthermore, Shen et al. [75] designed a WSN for structural health monitoring. The system was highly stable, durable and robust to electromagnetic interference. Similarly, the WSN was also used in structural health monitoring for the new headquarter of Shenzhen Stock Exchange (NHSSE) [76]. As one of the largest cantilever structures in the world, NHSSE’s strain and deflection data are vital for structural health. 224 sensors were installed to measure the strain, deflection and overall dynamic responses. The WSN is low-cost and easy to deploy and maintain. Along with various sensors, it is versatile enough to satisfy different requirements. Various magnitudes of interest such as acceleration, displacement, stress, temperature, etc., can be acquired simultaneously in a real-time manner. In addition, the system could provide an effective way for data management and thus save time and labor.

### 4.5. Safety Training and Education

Safety training and education (ST&E) for workers has a positive effect on enhancing safety management and reducing the occurrence of accidents. More concretely, based on the experience of construction industry experts, the benefits of ST&E for workers are as follows: (a) promote safety consciousness, align the interests of workers with security purposes; (b) adjust production and conduct safety operation positively; (c) obey safety regulations and policies consciously; (d) actively check and eliminate hidden dangers; (e) identify hazards and dangerous zones in advance, master effective response measures to safety accidents. Compared with traditional safety training and education, the sensor-based technology provides a novel approach to implement it. Tao et al. [77] built a framework based on real-time positioning data collected by sensor-based technologies. Three case studies showed that the framework performed well in data recording, stream processing and visualization. It could simulate construction site, outdoor construction environment and workers’ training environment, which provided a good platform for workers’ ST&E. Similarly, Jaselskis et al. [78] used mobile cameras to obtain information of various construction scenes and transmitted it in the form of audio or video to the off-site managers and experts. By connecting remote and off-site supervision, it offered a good opportunity for workers’ off-site ST&E.

### 4.6. Highly Dangerous Operations Management

The construction of infrastructures such as tunnels and mines is a complicated process due to the unstable and unpredictable construction environment. Accurate positioning and real-time tracking of workers can not only serve for real-time safety monitoring of dangerous areas, but also provide a reliable means to rescue trapped people timely in accidents. Lin et al. [79] developed a real-time tunnel location-based services (LBS) system (LBS) based on a WLAN. Taking advantage of fingerprint coding algorithms and an artificial neural network, it could estimate workers’ positions based on signal intensity with an accuracy of 3–5 m shown in the field experiments. It has been successfully applied to safety management of the world’s second largest hydropower project. Yuan et al. [80] used WLAN to build a high speed heterogeneous network which was able to adapt to a variety of harsh underground environment with low energy consumption, long operating life and high positioning accuracy.

As a big mining country, China has been embarrassed by the frequent mining accidents due to its lagging technology and weak security awareness. In recent years, along with the development of technology and the promotion of safety management, a great improvement has happened in China in mining safety. A large number of methods based on location sensors have been proposed for real-time underground positioning in coal mines. Some studies are mature and have resulted in the establishment of practical systems. For example, RFID-based underground positioning has been verified by experiments and practice [81,82,83,84]. The system has been promoted in a wide range. Similarly, researchers and safety managers have also shown their interest in Zigbee-based location system, which is supplemented by other locating sensor due to its short range [85,86,87,88].

The safety management of highly dangerous operations inspires construction safety management. The advancements in construction techniques and management have greatly reduced the rate of severe construction accidents, however, monitoring the position of workers in dangerous workplaces is never a waste of resources. In case of abnormal situations or accidents, the managers can acquire timely and vital information and take effective measures to reduce the impact of accidents. On-site rescue operations can save time and avoid secondary accidents.

## 5. Directions for Future Work

Several directions for future research can be identified:

(1) Integrated sensor-based technologies

Single sensor-based technology cannot satisfy the ever-increasing difficult requirements introduced by the needs of construction safety management. Therefore, the integrated development of multiple sensor-based technologies has become a trend. Specifically, not only can it compensate for the drawbacks of utilizing one single sensor, but also improve the accuracy of the collected information. For example, combining GPS and other wireless locating techniques usually yields better positioning results. The former is used to locate outdoor people and machinery equipment, while the latter is applied to acquire accurate positioning data of construction materials, resources and people in complex indoor environments. Meanwhile, multiple sensor-based technologies can share the responsibility for massive information collection, reduce the failure rate of the whole system and improve the overall stability and flexibility.

(2) Expansion in information dimensionality and data utilization

At this stage, the application of sensor-based technology is subject to strong restrictions so that the collected information of construction environment has lower dimensionality. For example, the original data covers either the dimension of time or dimension of space, but the manager needs to make decisions based on data providing both temporal and spatial information; the original data only provides a worker’s physical position, but the manager needs the worker’s physiological information to determine whether he or she is safe. The collected information typically covers only a few dimensions, which leads to the awkward situation that safety management cannot be reliably conducted due to incomplete information. At the same time, the capacity of processing the collected information is not satisfying. Only a small part of data can be identified and utilized, remaining the most part ignored directly. How to expand information dimension and increase the data utilization is of critical importance in the application of sensor-based technology in construction safety management.

(3) Exploration in various fields

The application of sensor-based technology in construction safety management covers the whole process of construction projects, including planning, design, construction and operation. Until recently, most research focused on the project construction phase, mainly based on a simulated construction environments. The systems, frameworks, models and algorithms proposed have not been fully tested in real construction environments, therefore, the actual performance is unknown to some extent. Only a few studies have investigated the cost-benefit of sensor-based technology, thus it is not validated to promote its application in construction safety management. In general, actively seeking the exploitation and exploration in various fields of sensor-based technology application is one of the current development trends in construction safety management.

(4) Hardware cost control and data processing simplification

As mentioned before, the development of sensor-based technology in construction safety management is restricted by two aspects: hardware and software. It is vital to control hardware costs within a reasonable level and simplify data processing of software. One direct consequence of cost control is the lack of accuracy in measurements. Though, to a certain extent, it can be compensated by using more complex algorithms, it is generally impractical to complete huge computation tasks at construction sites, which will inevitably increase the hardware costs as well. Taking the case of identification of workers’ unsafe behavior, a cheap and mature technique with simple layout (e.g., vision-based sensing) usually results in a complicated subsequence. Specifically, the contour extraction, noise reduction and posture recognition relies on algorithms and models with high complexity or huge computation burden, which are not practical to perform at construction sites. Peddi et al. [89] developed an algorithm to track workers, extract their contours and recognize their postures based on video information; Liu et al. [90] adopted the 3D skeleton extraction method with video stream; Han [91] proposed an unsafe behavior detection framework for workers on the basis of vision analysis; Park et al. [92] applied a video frame detection method to recognize workers’ unsafe behavior of not wearing safety helmets.

On the other hand, the one-sided pursuit of simplification in data processing usually requires highly complex sensors and data transmission systems. These devices are expensive and not easy to install and maintain. Staying with the unsafe behavior identification example, Han et al. [93] proposed a dimension reduction method for high dimensional motion analysis based on KPCA (Kernel Principal Component Analysis) to recognize workers’ unsafe behavior. The system required the installation of multiple motion sensors at workers’ different joints which was impractical in construction environment. Therefore, seeking the balance between hardware feasibility and software simplicity is of great importance in the healthy development of sensor-based technology in construction safety management.

(5) Smartphone-based interactive safety management platforms

Smartphone are an integrated platform containing multiple sensors. In recent years, smartphones have remained affordable because of their substantial popularity and price advantage, as a result of large-scale production. Smartphones have the potential to become an information management platform by virtue of their data processing ability and overall performance. A more desirable feature is that smartphones are open source systems with a modern software development environment, which is beneficial in information collection and data processing.

Nowadays, some researchers have shifted their focus of research to the application of smartphones and further exploited their features to build interactive platforms for construction safety management. Lim et al. [94] detected changes in workers’ energy release through the three-axis accelerometer in smartphones. With the help of an artificial neural network algorithm, the cause of energy release (slip or trip) was determined. In the aspect of classification and prevention of high altitude falling accidents in building construction, Kim et al. [95] designed a real-time position and risk automatic identification system based on smartphones and BIM technology. Their field experiments showed that the system could enhance the ability of environment monitoring on construction sites and had a potential in identification of high altitude falling accidents. Dzeng et al. [96] utilized smartphones to detect and identify the signs of high altitude falling accidents. Their tests showed that an acceleration-based algorithm could detect the signs of high altitude falling accidents effectively and accurately and it would not suffer from interference by construction activities.

Overall, a smartphone provides a good balance between hardware costs and software adaptability. It will play an important role in construction safety management as an interactive safety management platform in the foreseeable future. The significant advantages of smartphones are as follows: (a) the collected data includes multi-dimensional information including time, space, etc.; (b) they can acquire data in real time; (c) their high popularity makes them suitable for collection of massive amounts of information on a large scale. Therefore, a novel interactive safety management platform based on smartphones is expected to provide new ideas for the application of sensor-based technology in construction safety management.

## 6. Conclusions

This paper has provided a comprehensive and systematic review of sensor-based technology applied in construction safety management from 2005 to 2016. In order to give an objective evaluation of the current research status and future trends, a two-stage literature selection method was applied in this research. Taking Web of Science as a primary source and Engineering Index as a supplement, the research identified 93 papers in the preset topics and formed a database. The year profile of publications, application trends of sensor-based technology and distribution of research topics were analyzed statistically. Furthermore, the trends of the research topics and application potential were discussed in the analysis.

Sensor-based technology consists of location sensor-based technology, vision-based sensing and wireless sensor network. The paper summarized the positioning accuracy of locating sensor-based technology including GPS, RFID, WLAN, UWB, Zigbee and ultrasound, and gave a brief introduction to vision-based sensing and wireless sensor network. By analyzing the selected 93 papers, research topics were classified as accident forewarning systems, safety route prediction and planning, integrated safety management, structure health monitoring, safety training and education and highly dangerous operations management. Meanwhile, the application status of sensor-based technology in the above fields was introduced objectively and systematically. Based on the previous achievements, the paper identified the research gaps, pointed out future research directions and showed the prospects of future development.

In general, single sensor-based technology is not directly applicable in construction safety management. Only the integration of multiple techniques is capable of meeting the ever-increasing requirements. Since the hardware and software are the essential parts of sensor-based technology, a balanced realization will achieve better and faster progress of sensor-based technology in construction safety management. As an integrated platform of multiple sensor-based technologies, in recent years, the price of smartphones have become attractive because of their substantial popularity and price advantages resulting from large-scale production. In addition, due to the data processing ability and overall performance of smartphones, they have the potential to become an information management platform based on an open source system, which is beneficial in information collection and data processing. Generally, smartphones take both the hardware costs and the software compatibility into consideration and balances their relation in their evolution. Beyond that, compared with other sensor-based technologies, smartphones have some significant advantages, making them an optimal choice for both effective data acquisition solutions and portable data processing platforms. Considering the environment of construction sites and the information requirements of safety management, in the foreseeable future, smartphones will have an increasing role to play in interactive construction safety management. In conclusion, there is still a long way to go for the development of sensor-based technology construction safety management from theoretical research to practical application. Nevertheless, without a doubt, this field of application has great potential and a bright future.

## Figures and Tables

**Table 1:** Accuracy of locating sensor-based technologies.

Locating Sensor-Based Technologies	Accuracy from Publications (Best Result)
GPS	2.15–4.36 m [4]
RFID	0.86–2.6 m [29]
WLAN	1.5–4.57 m [11]
UWB	0.3 m [30]
Zigbee	0.76 m [17]
Ultrasound	0.04 m [15]

**Table 2:** Comparison of the three sensor-based technologies’ adaptability.

Sensor-Based Technology		Algorithm Complexity	Layout Complexity	Construction Environment Limitation
Locating sensor-based technology	GPS	Low	Low	(1) Suitable for outdoor environment
	UWB	Low	Moderate	(1) Accuracy affected by the arrangement of signal transmitters and receivers (2) Signals blocked or interfered by obstacles (3) Signals interfered by metal objects
	Zigbee	Low	Moderate	
	RFID	Low	Moderate	
	WLAN	Low	Moderate	(1) Signals blocked or interfered by obstacles
	Ultrasound	Low	Moderate	(1) Signals blocked or interfered by obstacles (2) Signals interfered by metal objects
Vision-based sensing technology		High	Moderate	(1) vulnerable to the impact of surrounding environment, such as lighting condition and background color
Wireless sensor network		Moderate	High	(1) Signals blocked or interfered by obstacles or other electronic signals in network communication (2) Difficult to solve the energy supply problems

**Table 3:** Commonly-used algorithms to improve construction safety management.

Category		Algorithm
Localization		Triangulation
		Angle of Arrival (AoA)
		Received Signal Strength Indication (RSSI)
		Time of Arrival (ToA)
		Time Difference of Arrival (TDoA)
		Roundtrip Time of Flight (RToF)
		Received Signal Phase Method (RSPM)
Object Tracking		Feature-based Tracking Algorithm
		Model-based Tracking Algorithm
Image Matching		Area-based Matching Algorithm
		Feature-based Matching Algorithm
		Computer Vision
Noise & Error Removal		Kalman Filter
Classification	Machine Learning	K-Nearest Neighbor Classification (KNN)
		Decision Tree
		Support Vector Machine (SVM)
		Naive Bayes Classification
		Artificial Neural Network (ANN)
		Convolution Neural Network (CNN)

**Table 4:** Main applications of sensor-based technology in construction safety management.

Applications in Construction Safety Management		Sensor-Based Technology	Application Significance
Accident prevention		RFID, UWB, Zigbee; Sensors and WSN	Prevent collision accidents of heavy machinery equipment
Safety Design	(1) Route prediction and planning for machinery equipment	RFID, UWB; Vision- based sensing	Predict trajectory of machinery equipment and workers
	(2) Route prediction and planning for workers		
Hazard identification	(1) Classification and identification of dangerous zones	RFID, UWB, Ultrasound; Vision-based sensing; Sensors and WSN	Prevent workers entering into dangerous zones unintentionally; Identify workers’ unsafe behavior
	(2) Identification of workers’ unsafe behavior		
Integrated safety management	(1) Quality inspection and management of construction material and resource	GPS, RFID, UWB, ultrasound; Vision-based sensing; Sensor and WSN	Inspect construction materials and resource quality; Provide fresh ideas for workers’ safety management
	(2) Health and safety management of workers		
Structural health monitoring		Sensor and WSN	Monitor structural health
Safety training and education		GPS, RFID; Vision-based sensing	Provide a new platform for workers’ safety training and education
Accident forewarning system		GPS, RFID, UWB, Zigbee, ultrasound; Sensor and WSN	Explore to build forewarning system for collision accidents of machinery equipment and high altitude falling accidents
Highly dangerous operations management		RFID, UWB, Zigbee, WLAN	Lay a solid foundation for real-time safety monitoring for dangerous zones
